# Spatial Epidemiology of Salmonellosis in Florida, 2009–2018

**DOI:** 10.3389/fpubh.2020.603005

**Published:** 2021-01-11

**Authors:** Xiaolong Li, Nitya Singh, Elizabeth Beshearse, Jason L. Blanton, Jamie DeMent, Arie H. Havelaar

**Affiliations:** ^1^Department of Environmental and Global Health, Emerging Pathogens Institute, University of Florida, Gainesville, FL, United States; ^2^Animal Sciences Department, Emerging Pathogens Institute and Food Systems Institute, University of Florida, Gainesville, FL, United States; ^3^Food and Waterborne Disease Program, Florida Department of Health, Tallahassee, FL, United States; ^4^Bureau of Public Health Laboratories, Florida Department of Health, Jacksonville, FL, United States

**Keywords:** *Salmonella enterica*, epidemiology, spatial-temporal trends, seasonality, serotypes

## Abstract

Non-typhoidal *Salmonella enterica* infections cause a high disease burden in the United States with an estimated 1.2 million illnesses annually. The state of Florida consistently has a relatively high incidence compared to other states in the United States. Nevertheless, studies regarding the epidemiology of nontyphoidal salmonellosis and its spatial and temporal patterns in Florida were rarely reported. We examined the spatial and temporal patterns of 62,947 salmonellosis cases reported to FL Health Charts between 2009 and 2018. Dominant serotypes circulating in Florida were also explored using whole genome sequencing (WGS) based serotype-prediction for 2,507 *Salmonella* isolates sequenced by the Florida Department of Health during 2017 and 2018. The representativeness of laboratory-sequenced isolates for reported cases was determined by regression modeling. The annual incidence rate of salmonellosis decreased from 36.0 per 100,000 population in 2009 to 27.8 per 100,000 in 2016, and gradually increased in 2017 and 2018. Increased use of culture-independent testing did not fully explain this increase. The highest incidence rate was observed in children, contributing 40.9% of total reported cases during this period. A seasonal pattern was observed with the incidence peaking in September and October, later than the national average pattern. Over these 10 years, the Northeast and Northwest regions of the state had higher reported incidence rates, while reported rates in the Southeast and South were gradually increasing over time. Serotypes were predicted based on WGS data in the EnteroBase platform. The top-five most prevalent serotypes in Florida during 2017–2018 were Enteritidis, Newport, Javiana, Sandiego and Braenderup. The highest percentage of isolates was from children under 5 years of age (41.4%), and stool (84.7%) was the major source of samples. A zero-inflated negative binomial regression model showed that the reported case number was a strong predictor for the number of lab-sequenced isolates in individual counties, and the geospatial distribution of sequenced isolates was not biased by other factors such as age group. The spatial and temporal patterns identified in this study along with the prevalence of different serotypes will be helpful for the development of efficient prevention and control strategies for salmonellosis in Florida.

## Introduction

Nontyphoidal salmonellosis is a common foodborne illness caused by a great diversity of *Salmonella enterica* serotypes with typical symptoms of diarrhea, abdominal cramps, vomiting and fever (1). In most cases, salmonellosis is a self-limiting illness and does not require medical treatment or hospitalization, but it is still problematic, especially among specific populations like children under 5 years-old, the elderly, and immunocompromised individuals. Humans get infected with *Salmonella* primarily by eating contaminated food (2). *Salmonella* infection poses a considerable disease burden globally, with an estimate of 93.8 million nontyphoidal *Salmonella* gastroenteritis cases occurring each year. Of these, 80.3 million cases were food-related (3). In the United States, *Salmonella* species are the second leading cause of foodborne illness. According to the estimates by Centers for Disease Control and Prevention (CDC), there are about 1.2 million illnesses, 23,000 hospitalizations, and 450 deaths associated with *Salmonella* infection annually (4). In the United States and other developed countries, a major portion of *Salmonella* illnesses has been attributed to food sources (5, 6). Seeded vegetables, chicken, fruits, pork, beef, eggs and other produce are food categories which contributed to more than 75% of outbreak-related *Salmonella* illnesses in the US (6). Previous source attribution studies conducted in other parts of the world also suggested that contaminated food including eggs, broiler chickens, and pigs were the top sources for human salmonellosis and outbreaks (5). In addition to the food pathway, water (e.g., recreational water/drinking water), person-to-person, animal contact and environmental exposure, such as contact with contaminated mud or soil, also contribute to transmission of *Salmonella* infection. A new study from the US suggests these pathways may contribute more than previously estimated (7).

The relative contribution of each transmission pathway varies among serotypes. Luvsansharav et al. developed a new measure to determine the foodborne relatedness of serotypes by calculating the ratio of proportion of laboratory-confirmed infections caused by each serotype to the proportion of foodborne illnesses associated with each serotype in the recorded outbreaks. Serotypes Saintpaul, Heidelberg and Berta showed the highest foodborne relatedness among the top 20 serotypes in the US; whereas, serotypes Mississippi, Bareilly, and Paratyphi B variant L(+) tartrate(+) had the lowest relatedness (8). Infection with *S*. Newport, the third most common serotype in the US, has been considered to be linked with both the food pathway and environmental sources, such as creek water and sediment (9). While infections from uncommon serotypes are more likely to be attributed to environmental factors and wild animals which are thought to primarily contribute to the variations in serotype diversity (10, 11). Moreover, some serotypes were frequently linked to specific food vehicles attributed to foodborne outbreaks in the US. For example, the salmonellosis outbreak data from CDC's Foodborne Disease Outbreak Surveillance System indicated that 81% of outbreaks with identified source of eggs were associated with *S*. Enteritidis between 1998 and 2015 (12).

Nontyphoidal *Salmonella* has been the leading cause of enteric illness reported in Florida which has consistently been one of the states with the highest incidence rates of salmonellosis in the United States (13). Nevertheless, studies regarding the epidemiology of nontyphoidal Salmonellosis and its spatial and temporal patterns in Florida were rarely reported.

As per the revisions to Rule 64D-3.029 Florida Administrative Code effective in October 20, 2016, laboratories in the state are required to send isolates or specimens of organisms responsible for reportable diseases including salmonellosis to the Florida Bureau of Public Health Laboratories (BPHL) for confirmation or additional characterization (14). Pulsed-field Gel Electrophoresis (PFGE) used to be considered the “gold standard” DNA fingerprinting method used by CDC within PulseNet. However, PulseNet transitioned from PFGE-based serotyping to a whole genome sequencing (WGS) -based method in 2019. BPHL has begun WGS in 2017 and has ceased the identification of PFGE for *Salmonella* isolate typing across the state of Florida and has started increasing the proportion of isolates sequenced with the WGS method, which provides the basis for examining the dominant *Salmonella* serotypes in Florida with WGS data available for 2017 and 2018.

In this study, we aimed to understand the incidence rate, temporal and geographical distribution and the demographics of reported salmonellosis cases in Florida from 2009 to 2018; to elucidate the dominant serotypes circulating in Florida based on the available WGS data in 2017 and 2018 and to determine the representativeness of laboratory-sequenced isolates for reported cases in Florida.

## Materials and Methods

### Data Sources and Study Setting

The state of Florida is located in the southeast of the United States. It consists of 67 counties with an estimated population of 21,477,737 in 2019 (https://www.census.gov/quickfacts/FL, accessed November 2, 2020).

The definition of salmonellosis case was according to the Florida Department of Health's Surveillance Case Definitions for Select Reportable Diseases in Florida (15). Case data of salmonellosis (including outbreak-related but not travel-related cases) in Florida were downloaded from http://www.flhealthcharts.com/ (accessed October 31, 2019) and included reported incidence and incidence rates and demographic information such as age and gender, from January 2009 to December 2018 in 67 counties of Florida. National data on salmonellosis incidence rate were obtained from the National Notifiable Diseases Surveillance System (https://wwwn.cdc.gov/nndss/, accessed May 15, 2019) for comparison.

Annual estimates of county resident population by age and the estimated population by ethnicity in Florida were obtained from the U.S. Census Bureau (https://data.census.gov/cedsci/, accessed October 31, 2019). As per ethical requirements for the purpose of visualization, counties with a population <20,000 were merged with other counties based on contiguous and comparable geographical locations (coastal or inland) and median household income, a total number of 58 (merged) counties were used in this study (16).

The land area data used for calculation of population density were derived from the Census 2010 summary file (https://www.census.gov/data/tables/2010/dec/2010-summary-file-1.html, accessed October 31, 2019).

Median household income for each county in Florida between 2017 and 2018 was obtained from the U.S. Census Bureau's Small Area Income and Poverty Estimates (SAIPE) program (https://www.census.gov/programs-surveys/saipe.html, accessed October 31, 2019).

Metadata of sequenced isolates from the BPHL Bionumerics database and the FDOH Merlin database were merged by a surveillance epidemiologist at FDOH and deidentified data were shared with UF under a data sharing agreement, resulting in a set of 2,507 complete records, 1,353 isolates from 2017 and 1,154 isolates from 2018. Sequencing of included isolates was performed at BPHL in Jacksonville who follows a standardized sequencing protocol and shares all sequenced data with CDC for surveillance purposes. These data are submitted to the NCBI BioSample database (https://www.ncbi.nlm.nih.gov/biosample/), under Bioproject number PRJNA230403 with limited metadata information. More detailed standardized protocol can be found in Supplementary Material.

All datasets were cleaned, organized and analyzed in the R statistical software version 3.6.0 (17).

### Epidemiological Analysis of Surveillance Data, 2009–2018

Annual incidence, incidence rate and monthly cumulative incidence from 2009 to 2018 in Florida were plotted to illustrate trends and seasonal patterns. The incidence rate was age-adjusted using direct standardization at both county and region level with the age distribution in the state of Florida as a reference (18). A 3-month moving average was used to smooth the monthly salmonellosis data to assess the overall trend. A series of thematic maps, based on age-adjusted incidence rate data at the county level, were used to analyze spatial and temporal distribution of salmonellosis in counties of Florida.

Median polish was used to explore the variation of salmonellosis age-adjusted incidence rate among six regions in Florida as defined by the FDOH Weekly Morbidity Statistics Report (19) and years and was represented by the following formula:

$$\begin{array}{l} y_{ij}\;=\mu +\alpha_{i}+\;\beta_{j}\;+\varepsilon_{ij} \end{array}$$

where *y*_*ij*_ is the response variable (incidence rate of salmonellosis) for row *i* and column *j*, μ is the common value (median), α_*i*_ is the row (region) effect, β_*j*_ is the column (year) effect and ε_*ij*_ is the residual (20).

The row/column effects reflect the influence of each level of region/year on the incidence rate of salmonellosis, while the residuals indicate how incidence rates of salmonellosis from different regions and in different years are distinct from each other after removing the median value and corresponding row and column effects.

### Analysis of Laboratory-Sequenced Isolates, 2017–2018

Incidence rates of laboratory-sequenced isolates were calculated at the county level for the years 2017 and 2018 separately. For 2017, 56 isolates were dropped (6 with missing zip code information, 16 with PO Box zip codes and 34 from out of Florida residents), and 1,297 isolates were available for analysis. For 2018, 38 isolates were dropped (12 with missing zip code information, 16 with PO Box zip codes and 10 from out of Florida residents), and 1,116 isolates were available for analysis. Five-digit zip code was recorded for each sequenced isolate. We assigned zip code areas crossing county boundaries to one county based on the geometry center of the zip code shapefile polygon in ArcGIS (ERSI, Redlands, CA).

Serotype prediction was performed using Salmonella *in silico* Typing Resource (SISTR ver.1) algorithm built-in the EnteroBase online server (https://enterobase.warwick.ac.uk/ accessed October 31, 2019) (21). Of 2,507 lab-sequenced isolate records, 1,709 records were available in the EnteroBase server and were used for serotype prediction in this study. SISTR version 1 was not able to resolve the serotypes for 68 isolates. We submitted these sequences to the developer group of SISTR (21), who resolved the serotypes for these samples using an advanced version of SISTR (version 2) which is not yet released on any public platform.

To investigate whether sequenced isolates were a non-biased sample of reported cases, a zero-inflated negative binomial regression model was used including other putative explanatory variables [population density (person per square miles), median household income, and the percentage of children under 5 years and adults over 65 years, respectively, in the overall population].

## Results

### Epidemiology and Demographics of Salmonellosis Reported Cases, 2009–2018

**Figure 1:**
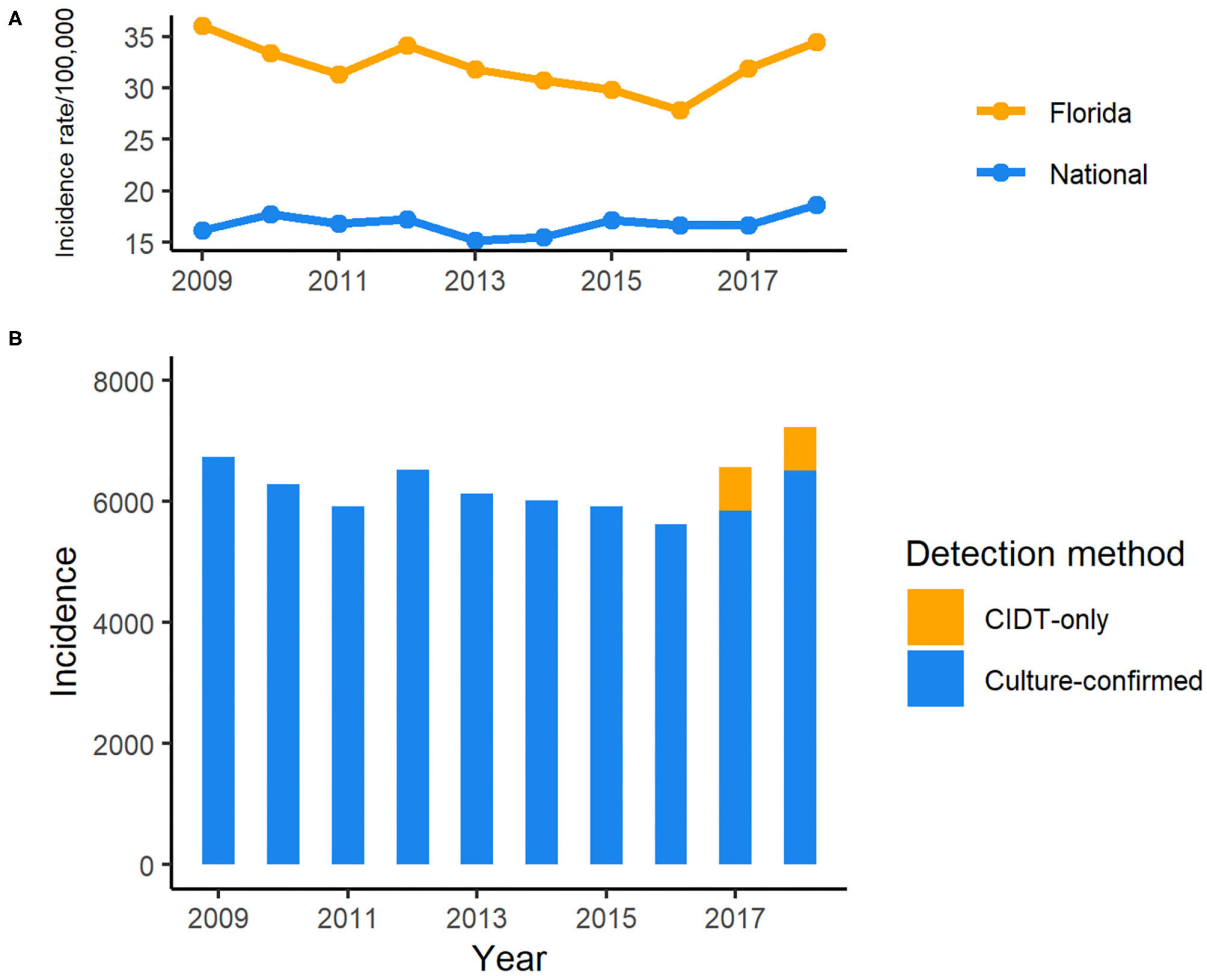
Incidence of reported cases of salmonellosis in Florida, 2009–2018 CIDT: Culture-independent diagnostic tests. **(A)** Incidence rate of salmonellosis in Florida and national average, 2009–2018; **(B)** Incidence of salmonellosis cases detected with different methods in Florida, 2009–2018.

During the period of 2009–2018, 62,947 salmonellosis cases were reported in Florida, accounting for 11.8% of the total number in U.S. cases for these 10 years. The annual incidence rates ranged from 27.8 to 36.0 per 100,000 population, much higher than the national average incidence (15.2–18.64/100,000; Figure 1). There was a decreasing trend in incidence rate, except in 2012 when a peak appeared, with the incidence rate dropping from 36.0 per 100,000 population in 2009 to 27.8 per 100,000 in 2016. However, the incidence rate of salmonellosis started to increase again in 2017. Of 6,557 reported cases in 2017, 720 cases were confirmed only by culture-independent diagnostic tests (CIDTs) that was included as one of the criteria in the revised case definition of salmonellosis in Florida since 2017. The incidence continued to rise in 2018, and 718 cases were confirmed by CIDT only.

**Figure 2:**
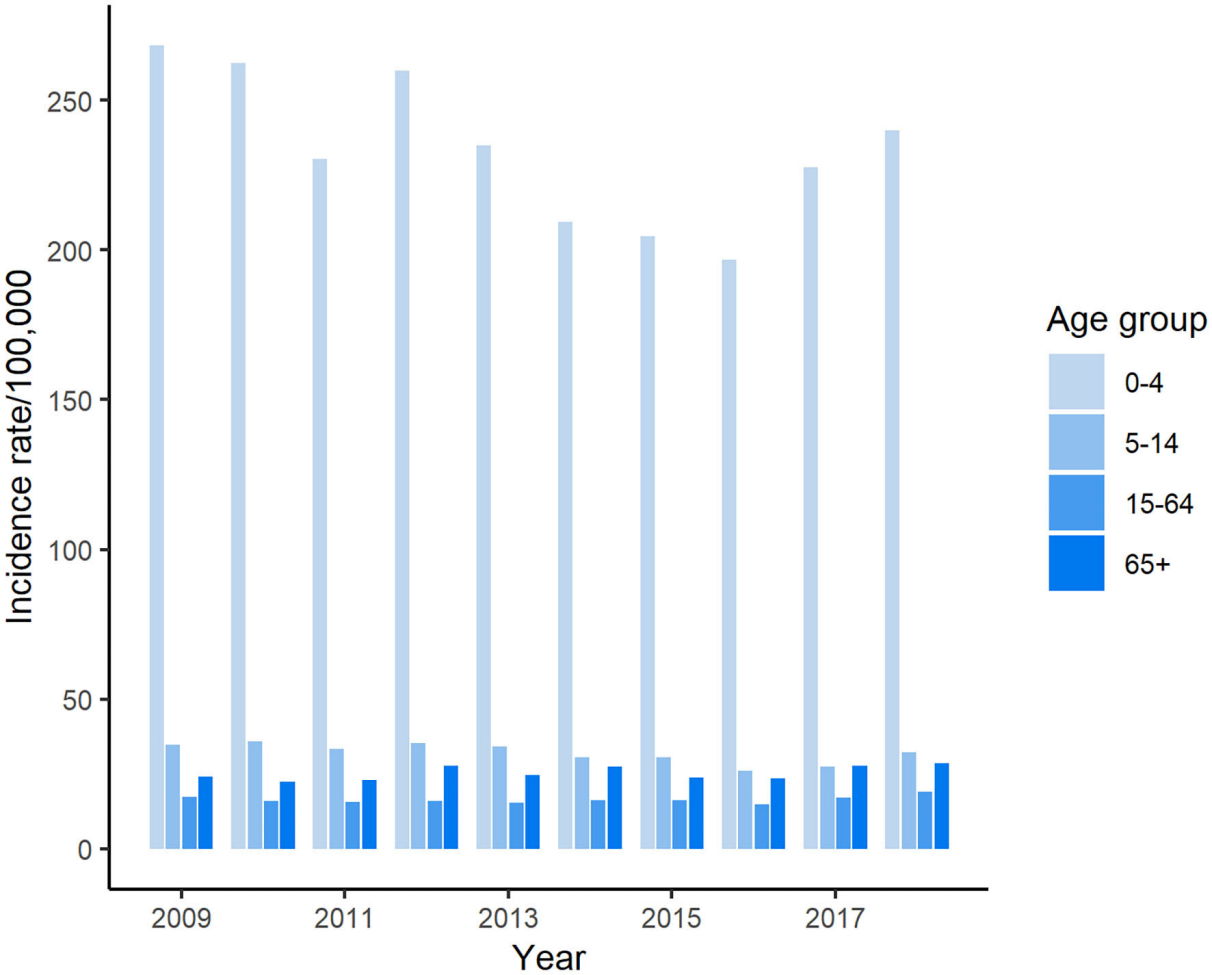
Age distribution of salmonellosis in Florida, 2009–2018.

The highest number of salmonellosis cases was detected in the under 5-years-old age group, which accounted for 40.9% (25,737/62,947) of the total number of reported cases in Florida during these 10 years. Among age groups, the incidence rate in the under 5-years-old age group reached above 250 per 100,000 population in some years, which was more than 7 times higher than that in any other age groups (Figure 2). The average incidence rate of salmonellosis in 15–64-years-olds was the lowest of all age groups. The trend of incidence rate among children under 5 years of age displayed a similarity with the overall pattern of all reported cases during the decade, decreasing from 2009 through 2016 and then ascending again from 2017 onwards.

### Seasonal Pattern

**Figure 3:**
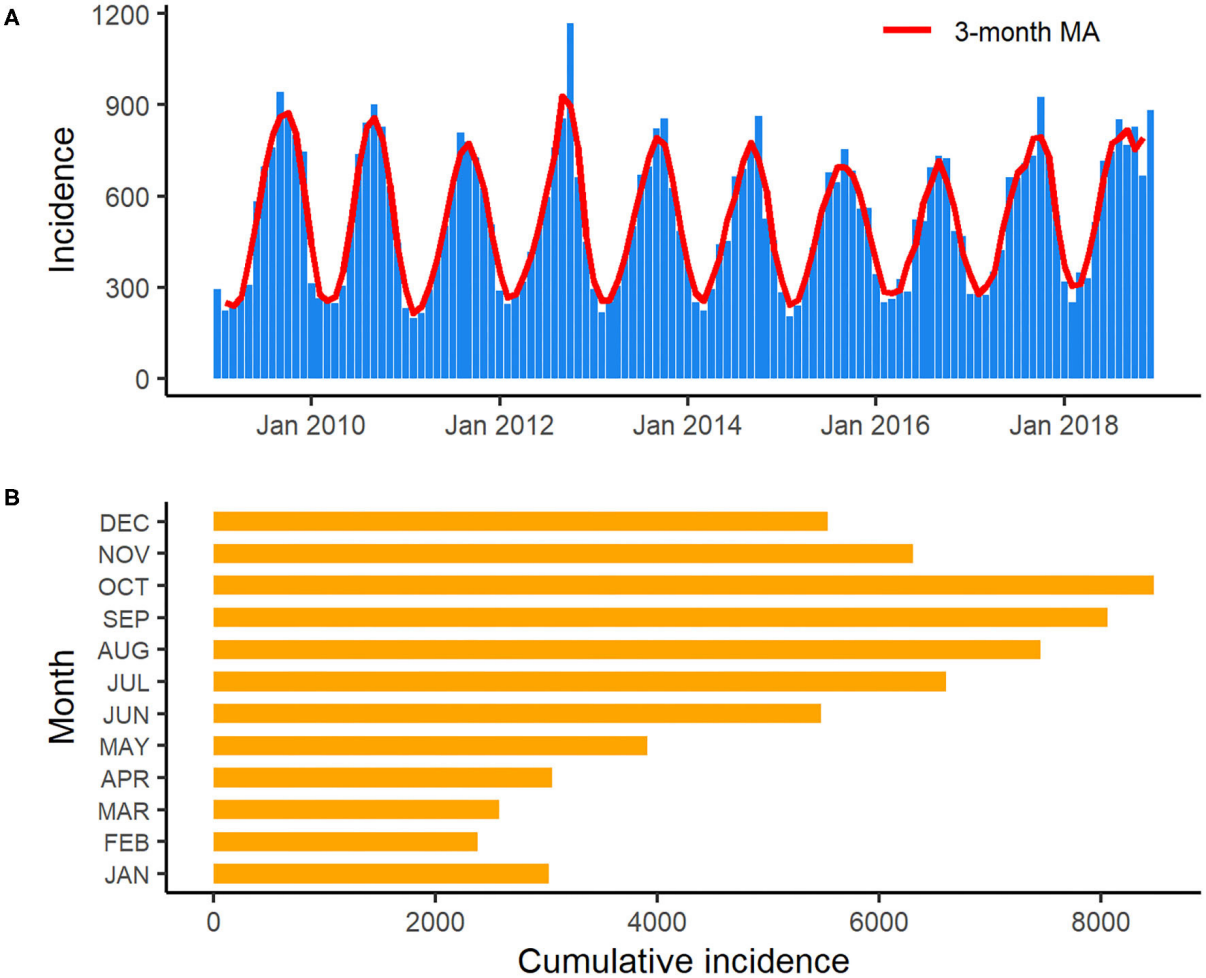
Monthly incidence and 3-month moving average of salmonellosis in Florida from January 2009 to December 2018 **(A)**: 3-month moving average smoothed monthly incidence of salmonellosis in Florida from January 2009 to December 2018; **(B)**: Cumulative incidence of salmonellosis for each month in Florida over the years 2009–2018.

An obvious seasonality was observed from the distribution of salmonellosis cases from January 2009 to December 2018 (Figure 3). Salmonellosis occurred throughout the whole year in Florida, but the incidence gradually rose through spring and summer and peaked in September and October. After October, the incidence decreased notably, and the lowest incidence was typically observed in February. A total of 26.3% (16,547/62,947) of salmonellosis cases occurred in September and October on aggregate in the decade. Data from the Laboratory-based Enteric Disease Surveillance for 2006–2015 show that the peak at national level typically occurred in August, i.e., 1–2 months before the peak in Florida. The lowest national incidence was typically observed in February, similar to Florida (22).

### Geographical Distribution of Reported Salmonellosis Cases

**Figure 4:**
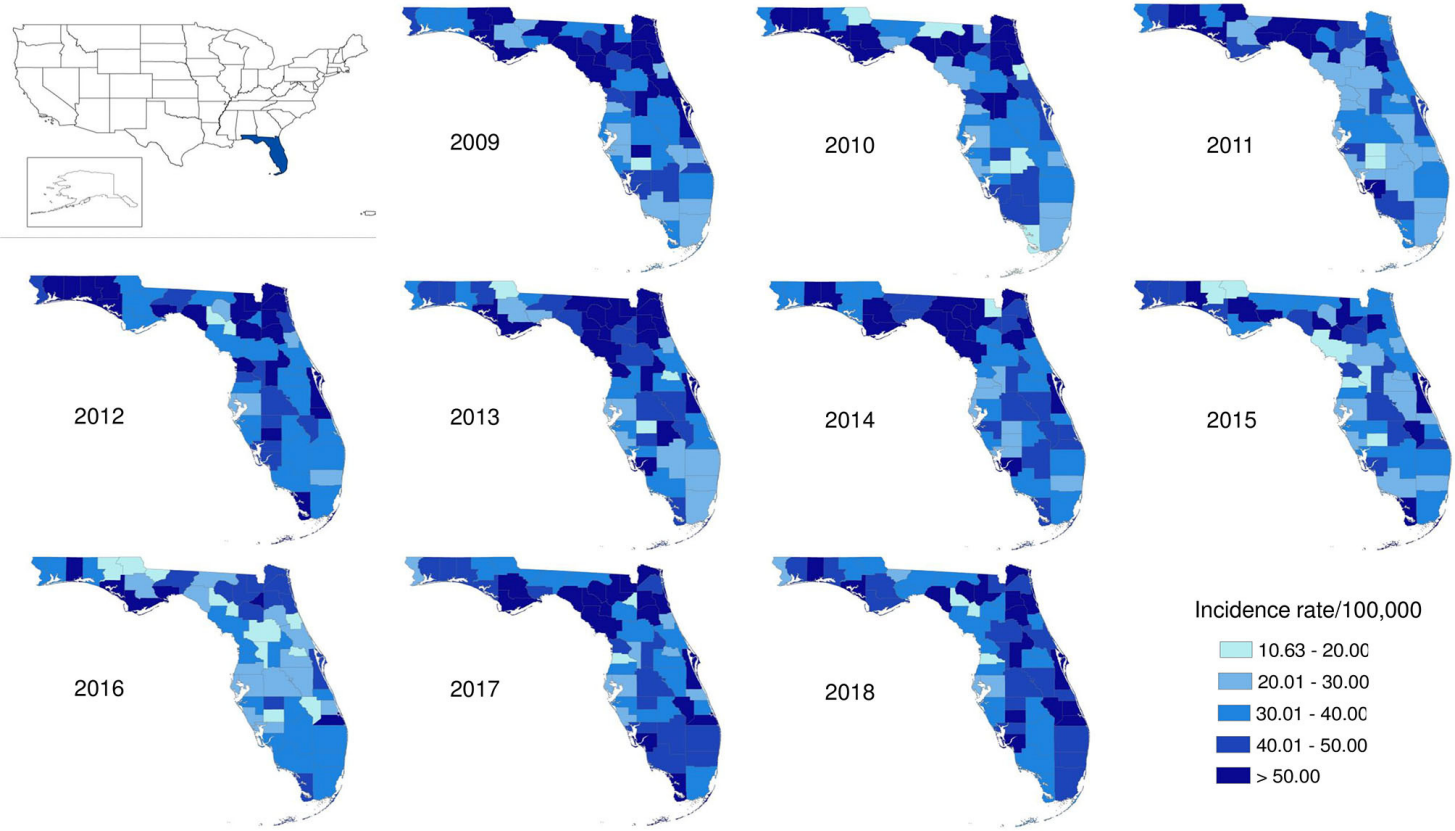
Geographical distribution of salmonellosis age-adjusted incidence rate at county level in Florida, 2009–2018.

The salmonellosis cases occurred in almost every county in Florida throughout 2009–2018 (Figure 4). The age-adjusted incidence rate in many counties could be as high as 50–60 per 100,000 population whereas in some years, it even went up to 80–90 per 100,000 population.

**Figure 5:**
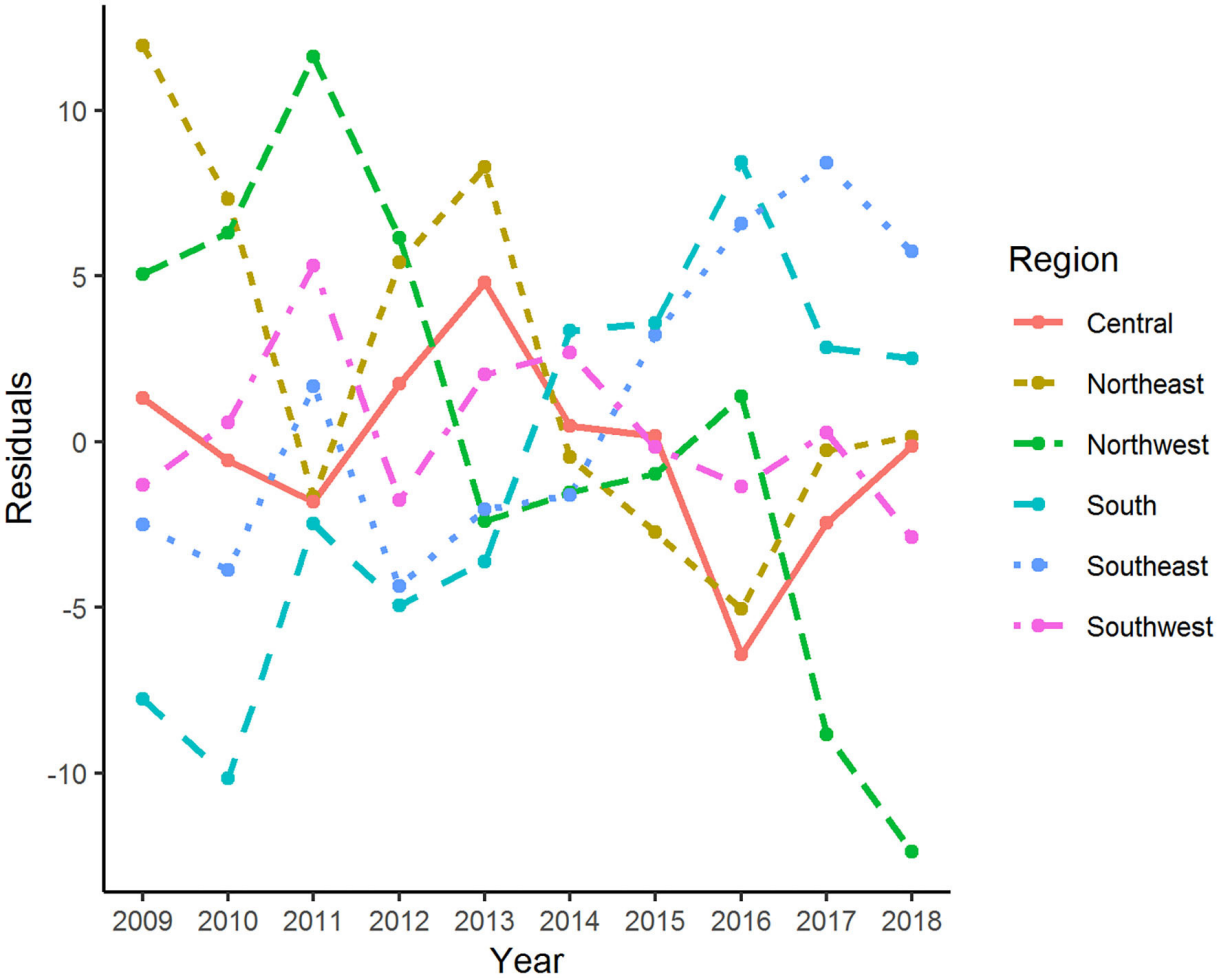
Residuals of age-adjusted incidence rate of salmonellosis for various regions and years after median polish.

The age-adjusted incidence rate of reported cases by region as used in the Florida Department of Health Weekly Morbidity Statistics Report is presented in Table 1. On average, the Northeast and the Northwest regions had the highest incidence rate with 47.0 per 100,000; while the Southwest and the Southeast regions had the lowest. To examine the variation of incidence rates between regions across these 10 years, Tukey's median polish analysis was implemented with the data listed in Table 1. The overall row (region) effect showed that the Northwest region had the highest incidence rate of salmonellosis with 10.9 per 100,000 higher than overall median, respectively; while the Southeast region had the lowest incidence rate at 4.3 per 100,000 below the overall median. Similarly, the effects from years (column effect) showed a higher incidence rate of salmonellosis in 2018 (with an effect value of 5.3) and lower incidence rates in 2015 (with an effect value of −3.7). A final residual table indicating the residuals of incidence rate for each region in a specific year after the row medians and column medians being removed iteratively was obtained and plotted as shown in Figure 5. It suggested that, although the incidence rates were higher in the Northeast and Northwest region than any other regions at the beginning of this decade, there was a decreasing trend on incidence rate observed in these two regions. On the contrary, the incidence rates in the Southeast and South regions were continually increasing during this time period.

**Table 1:** Age-adjusted incidence rates of salmonellosis in Florida by region, 2009–2018.

**Region**	**2009**	**2010**	**2011**	**2012**	**2013**	**2014**	**2015**	**2016**	**2017**	**2018**	**Average**
Northwest	53.0	52.7	54.6	56.2	43.0	45.1	41.9	44.4	39.3	39.5	47.0
Northeast	58.0	52.0	39.5	53.7	51.9	44.4	38.3	36.2	46.0	50.2	47.0
Central	40.4	37.0	32.3	43.0	41.4	38.3	34.2	27.8	36.8	42.9	37.4
Southwest	32.2	32.6	33.9	34.0	33.1	35.0	28.3	27.3	34.0	34.6	32.5
Southeast	30.2	27.4	29.5	30.6	28.2	29.9	30.9	34.5	41.3	42.5	32.5
South	27.2	23.3	27.6	32.2	28.9	37.1	33.5	38.6	38.0	41.5	32.8

### Descriptive Analysis of Lab-Sequenced Isolates, 2017–2018

The male: female ratio of sequenced isolates was 0.90:1.00 (Table 2). Based on the estimate by US Census Bureau in 2018, 26.1% of the Florida population were Hispanic and Latino, while 29.7% (617/2,076) of lab-sequenced isolates with known ethnicity were reported from this group between 2017 and 2018 (Table 2). Similar to reported cases, most lab-sequenced isolates were found in children under 5-years of age (Table 3).

**Table 2:** Demographics of 2,507 laboratory-sequenced salmonellosis cases with complete metadata in Florida, 2017–2018.

**Category**	**Number of cases**
**Gender**	
Male	1,186
Female	1,313
Unknown	8
**Ethnicity**	
Hispanic	617
Non-Hispanic	1,459
Unknown	431

**Table 3:** Age distribution of reported and laboratory-sequenced salmonellosis cases in Florida, 2017–2018.

**Age**	**Reported cases**	**Lab-sequenced isolates**
0–4 years	5,287 (38.4%)	1,037 (41.4%)^*^
5–14 years	1,381 (10.0%)	240 (9.6%)
15–64 years	4,778 (34.7%)	824 (32.9%)
65+ years	2,329 (16.9%)	405 (16.2%)
Unknown	0	1 (0.0%)
Total	13,775 (100%)	2,507 (100%)

* *Percent of cases with known age*.

#### Representativeness of Lab-Sequenced Isolates

**Figure 6:**
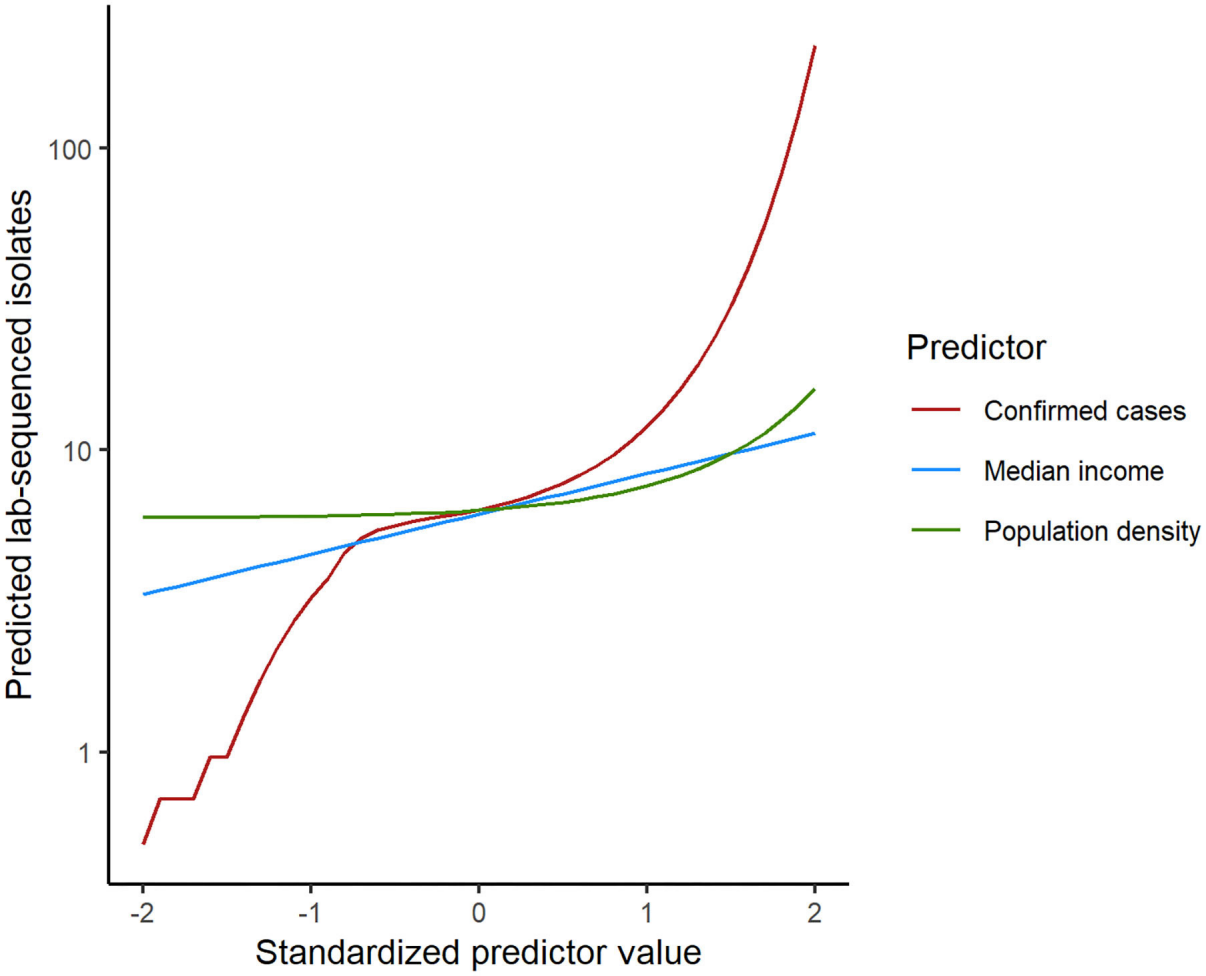
Comparison of the predictor performance in the zero-inflated negative binomial regression model for lab-sequenced isolates per county. Each predictor was standardized by subtracting the mean and dividing by the standard deviation. Model predictions were then calculated for each predictor separately while holding the other predictors at their median values.

To statistically test the representativeness of count of lab-sequenced isolates (spread of frequency counts included as Supplementary Figure 1) for the reported cases in counties of Florida, we fitted a zero-inflated negative binomial (ZINB) regression model with the number of reported cases from the surveillance system, population density (persons per square mile), median household income, the percentage of children under 5 years in the overall population and the percentage of adults over 65 years in the overall population as putative explanatory variables. The overall model performance statistics with statistically significant predictors are enumerated in Table 4, and the plot showing the model fit with observed data is included in Supplementary Figure 3. The Vuong test suggested that ZINB model was a significant improvement over the standard negative binomial (NB) model (detailed model diagnostics are enumerated in Supplementary Tables 1–3, and model fit with observed data is depicted in Supplementary Figure 2). In the logit model predicting excessive zeros, reported case number was the only (marginally) significant variable. ZINB model correctly predicted 19.79% excess zero for original proportion of 20.15%. The negative association suggested that the greater the reported case number, the less likely it was that no isolate was sequenced in a county. Reported case number, population density, and median household income were significant predictors in the negative binomial model predicting the count of the sequenced isolates. The prediction performance of these three predictors was further compared after standardization, demonstrating that the number of reported cases was the strongest predictor of the number of lab-sequenced isolates (Figure 6).

**Table 4:** Zero-inflated negative binomial regression model for lab-sequenced isolates per county.

**Parameter**	**Logit**			**Negative binomial**		
	**Estimate**	95% CI^*^	***P*-value**	**Estimate**	**95% CI**	***P*-value**
Intercept	2.66	(0.14, 5.17)	0.04	−15.80	(−24.49, −7.12)	<0.001
Reported case No.	−0.38	(−0.77, 0.01)	0.06	0.005	(0.004, 0.006)	<0.001
Population density				0.0004	(0.0002, 0.0006)	<0.001
Ln(median household income)				1.61	(0.81, 2.41)	<0.001
Dispersion	0.93					
Log-likelihood	−378.84; df = 7	<0.001				
AIC	771.68; df = 7					
BIC	791.97; df = 7					
RMSE^¥^ (LOO^§^ cross-validation average)	15.29					
Vuong test of ZINB versus NB^#^			<0.002			

* *95% confidence interval*.

# *ZINB, zero-inflated negative binomial; NB, negative binomial*.

¥ *Root Mean Squared Error*.

§ *Leave one out*.

#### Serotypes of Salmonella Isolates

Based on the 1,709 isolates that both were available in Enterobase and had complete metadata information available, a considerable variety of *Salmonella* serotypes was detected in Florida. Of the top-20 most frequently detected serotypes, 19 belonged to subspecies I *S. enterica* ssp. *enterica*, while one belonged to subspecies IV *S. enterica* ssp. *houtenae*. Serotypes Enteritidis, Javiana, Newport, Sandiego, and Braenderup were the top five most commonly detected serotypes (Table 5). Serotype Enteritidis had the highest incidence rate (0.90 per 100,000), followed by Newport and Javiana with incidence rates of 0.79 per 100,000 and 0.60 per 100,000, respectively. Of 1,709 isolates, ~46% belonged to less frequently detected serotypes (including 798 isolates that could not be assigned a serotype by SISTR).

**Table 5:** Top 20 *Salmonella* serotypes and corresponding sample sources in Florida based on the lab-sequenced salmonellosis cases between 2017 and 2018.

**Serotype**	**Number reported**	**Percent**	**Incidence/100,000**	Blood (%)^*^	**Stool (%)**	**Urine (%)**	**Unknown (%)**
Enteritidis	185	10.83	0.9	14 (7.6)	153 (82.7)	4 (2.2)	14 (7.6)
Newport	163	9.54	0.793	7 (4.3)	137 (84.1)	4 (2.5)	15 (9.2)
Javiana	123	7.2	0.598	3 (2.4)	106 (86.2)	7 (5.7)	7 (5.7)
Sandiego	123	7.2	0.598	15 (12.2)	99 (80.5)	6 (4.9)	3 (2.4)
Braenderup	91	5.32	0.443	0	79 (86.8)	6 (6.6)	6 (6.6)
Typhimurium	83	4.86	0.404	6 (7.2)	68 (80.7)	2 (2.4)	8 (9.6)
IV 50:z4,z23:-	77	4.51	0.375	9 (11.7)	55 (71.4)	4 (5.2)	9 (11.7)
Muenchen	60	3.51	0.292	0 (0)	54 (90)	1 (1. 7)	5 (8.3)
Rubislaw	59	3.45	0.287	3 (5.1)	49 (83.1)	2 (3.4)	5 (8.5)
Bareilly	54	3.16	0.263	0	50 (92.6)	1 (1.9)	3 (5.6)
Saintpaul	46	2.69	0.224	1 (2.2)	42 (91.3)	2 (4.4)	1 (2.2)
Montevideo	44	2.57	0.214	1 (2.3)	37 (84.1)	1 (2.3)	5 (11.4)
Poona	39	2.28	0.19	4 (10.3)	31 (79.5)	1 (2.6)	3 (7.7)
Oranienburg	38	2.22	0.185	5 (13.2)	28 (73.7)	1 (2.6)	4 (10.5)
Anatum	35	2.05	0.17	0	29 (82.9)	6 (17.1)	0
I 4,[5],12:i:-	35	2.05	0.17	0	1 (2.9)	34 (97.1)	0
Paratyphi B var. Java	25	1.46	0.122	0	22 (88)	1 (4)	2 (8)
Baildon	24	1.4	0.117	2 (8.3)	20 (83.3)	1 (4.2)	1 (4.2)
Thompson	23	1.35	0.112	2 (8.7)	21 (91.3)	0	0
Infantis	22	1.29	0.107	0	18 (81.8)	1 (4.6)	3 (13.6)
Other Serotypes	360	21.06	1.751	25 (6.9)	286 (78.3)	18 (5.0)	34 (9.7)
Serotype not available	798	46.69	3.882	32 (4.0)	705 (88.7)	4 (0.5)	54 (6.8)
All isolates	2,507	146.69	12.197	129 (5.2)	2,123 (84.7)	74 (3.0)	181 (7.2)

* *Source Percentages are row-wise for each serotype per sample group*.

#### Sample Sources and Serotypes

Sources of all 2,507 samples delivered to state laboratories were summarized by serotype, and the source distribution for the top 20 serotypes was displayed in Table 5. For all top 20 serotypes, the percentage of samples from stool ranged from 2.9 to 92.6% with an overall percentage of 84.7%. Overall percentage for blood was 5.2%; while, for serotypes Oranienburg (13.2%), Sandiego (12.2%), IV 50:z4,z23:- (Flint; 11.7%), and Poona (10.3%), at least 10% of isolates were from blood. Overall, 3.0% of isolates were from urine but 97.1% of isolates from serotype I 4,[5],12:i:-, a monophasic variant of Typhimurium, followed by 17.1% of Anatum isolates were from urine.

#### Geospatial Distribution of Lab-Sequenced Isolates

**Figure 7:**
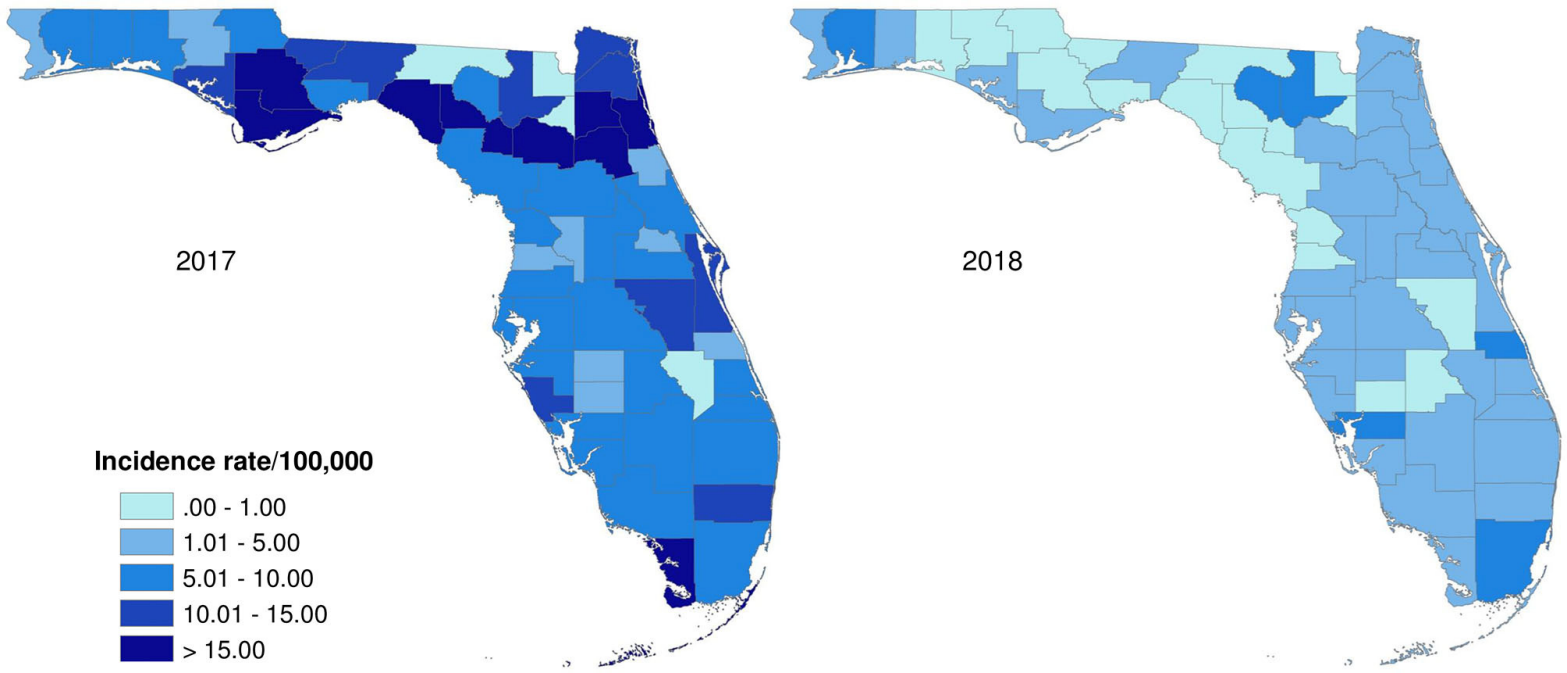
Age-adjusted incidence rate of lab-sequenced *Salmonella* isolates in Florida, 2017–2018.

The age-adjusted incidence rates of lab-sequenced *Salmonella* isolates in counties of Florida in 2017 and 2018 were illustrated in Figure 7. The origin of the *Salmonella* isolates appears to be distributed over the whole state. Generally, the incidence rates of lab-sequenced isolates were notably lower than that of reported cases, with most counties having an incidence rate <15 per 100,000 population. The incidence rates ranged from 0 to 41.1 per 100,000 population in 2017 and from 0 to 7.5 per 100,000 population in 2018. The incidence rates across counties were relatively higher in 2017 than 2018, and some counties with higher incidence rates were observed in the Northwest and Northeast regions in 2017.

## Discussion

Approximately 11,000–14,000 cases of enteric illness are reported each year in Florida, which makes Florida consistently one of the states with highest incidence rate of enteric diseases across the country (23). Among those, salmonellosis is the most common enteric illness in Florida with 6,000–7,000 cases reported annually. Generally, young children, especially children under 5 years old, are disproportionately affected by enteric illnesses (24, 25). Salmonellosis is not an exception. The CDC's Foodborne Diseases Active Surveillance Network (FoodNet) reported that children aged <5 years old had the highest incidence rate of salmonellosis (56.3 cases per 100,000 population) among age groups in 2015 (26). In Florida, the incidence rate in this age group ranged from 196.7 to 268.2 cases per 100,000 population during the years of 2009–2018. Such higher incidence rate in children may be partly due to the increased exposure to enteric pathogens through fecal-oral contaminations in different exposure settings (25, 27). Several risk factors like contact with pet reptiles and amphibians or live poultry (28, 29), riding in a shopping cart next to meat or poultry (30), and attending a day care center with a *Salmonella*-infected child have been linked to salmonellosis in children (30). It is not clear which proportion of *Salmonella* infections among children in Florida that can be attributed to these risk factors.

Despite the high reported incidence of salmonellosis in Florida, the *Salmonella* Annual Reports issued by CDC suggest a very low incidence rate in the state. For example, while the incidence rate of reported cases was 27.8 per 100,000 population in 2016 (the latest year for which the CDC report is currently available), the *Salmonella* Annual Report indicated an incidence rate of 5.07 per 100,000 population, the lowest in the country (22). Infectious disease surveillance results are reported from Florida to the National Notifiable Diseases Surveillance System (NNDSS), which accurately reflects the incidence reported by Florida. In contrast, the *Salmonella* Annual Reports are based on the Laboratory-based Enteric Disease Surveillance (LEDS) system, which collects data from state and regional public health laboratories. The seemingly disparate results are related to the limited capacity the state had to access and fully type isolates from all confirmed cases. Before 2016, when new requirements to submit isolates or samples were included in the Florida Administrative Code, BPHL only received between 1,900 and 2,500 specimens per year. The number of isolates submitted to BPHL has increased from about 4,500 in 2017 to about 7,200 in 2019. The current capacity of BPHL for sequencing *Salmonella* isolates is approximately 8,000. In 2016, Florida reported 1,007 isolates to LEDS, and this has increased to 5,810 in 2018. Thus, new regulations and the WGS workflow have greatly increased coverage of surveillance.

The distribution of salmonellosis cases in Florida displayed a strong seasonality, with cases spiking in late summer/early fall (September–October). Florida has a distinct climatic pattern with high average temperature and precipitation, especially in summer (the rainy season extends from May through October). Whether this specific climatic pattern contributes to the seasonality of salmonellosis, especially infections caused by several *Salmonella* serotypes related to non-food and environmental exposure needs to be further studied. It would be interesting to compare with seasonality of salmonellosis in other states with similar climates. More tropical storms taking place in the rainy season is another typical feature of Florida's climate, and nearly one third of these storms occurred in September. The influences of extreme weather events like hurricanes on food- and water-borne diseases have been previously reported (31, 32). The association between increasing salmonellosis cases and the effects of extreme weather events is also an interesting topic to address in the future study.

Though the incidence rate of salmonellosis has been continually high in Florida compared to the national level, it decreased by 23% in 2016 compared to 2009 but rebounded after 2016 and reached a peak in 2018. With the increasing use of CIDTs by clinical laboratories to diagnose enteric pathogens in recent years nationwide, CIDT was included in the criteria for suspected cases as a part of the expansion of salmonellosis case definition by Florida Department of Health in January 2017 (15). The CIDT positive-only cases contributed to 76 and 45% of the increase in total case number in 2017 and 2018, respectively, comparted with 2016 in Florida. Interestingly, the incidence rate of salmonellosis did not seem to be affected obviously by the wide adoption of CIDTs and remained relatively stable during these years (Figure 1). Nevertheless, Marder et al. reported the increasing use of CIDTs had complicated the interpretation of the enteric infection surveillance data (33). In some cases, the changes in laboratory testing practices had significantly increased the incidence rate of infections caused by enteric pathogens. For example, based upon the data from ten U.S. sites of FoodNet between 2013 and 2016, there was a significant increase in the incidence rates of both confirmed Shiga toxin-producing *Escherichia coli* (STEC) infection alone and confirmed or CIDT positive-only infections in 2016 compared with the average level of 2013–2015. For *Salmonella*, that increase was slight but not significant, although the proportion of clinical laboratories within the network of FoodNet that used CIDTs to detect *Salmonella* increased markedly to 14% in 2016 from <1% in 2013 (33). It is worth noting that the proportion of *Salmonella* infections identified by CIDTs without culture confirmation was only 8% within the ten sites of FoodNet, the lowest among other enteric pathogens monitored by FoodNet in 2016.

Underreporting is one of the major limitations for most surveillance systems of notifiable diseases; enteric disease surveillance system is not an exception. These surveillance data are heavily based upon notification of ill cases from health care providers and laboratories that can only capture affected cases who seek medical care after getting sick, whose specimens are tested by laboratory and results are being reported to the public health authorities. These laboratory-confirmed illnesses are only a fraction of the true disease burden across the nation. CDC estimates that for every case of salmonellosis reported to the national surveillance system, there are 29.3 cases in the population (4). Such estimates are not available for individual states. Regardless of the limitations, surveillance data are still helpful for determining the trend or patterns of disease occurrence and burdens (34).

Population data from floridahealthcharts.com do not account for annual fluctuations in population numbers affected by tourism and seasonal residence (snowbirds, seasonal agricultural workers). These areas are mainly found in Central and Southern Florida. Therefore, the incidence rates of salmonellosis may have been overestimated for these regions. Nevertheless, we did find that incidence rates were higher in the North and Northeast than in other regions. We did find an increasing incidence rate over the years in the Southern part of the state which might partly be explained by seasonal migration that is unaccounted for in population numbers.

Epidemiological studies have suggested that socioeconomic status (SES) indicators including educational attainment, household income, and poverty level are determinants that have been linked to the risk of salmonellosis at both individual and community level (35–40). Lower educational level of parents in a household, always along with a higher level of poverty, may be related to improper practice of food handling and poor personal hygiene, which contributes to the high occurrence of salmonellosis among their children living in this environment (36, 37). Therefore, adults with lower educational attainment should be the target population of food safety interventions in the future to decrease the incidence rate of salmonellosis among children.

Household income was another potential risk factor responsible for high incidence rate of salmonellosis. Previous studies have reported that families with higher income were more likely to seek healthcare with higher healthcare accessibility, eat outside, keep pets at home, and have more frequent international travels, which had been identified as risk factors that increase the exposure to enteric pathogens (36, 39). Besides, Varga et al. suggested that lower household income was also associated with increased *S*. Enteritidis infection in Canada, which can be partially explained by the universal access to provincially-founded healthcare in Canada, consumption of poor microbial quality food in low-income households, as well as the frequent violation of food safety procedures by retailers in the neighborhoods of low-income households (39). To better understand the role of household income in the increased risk of exposure to enteric pathogens, further serotype-specific analysis studying these associations needs to be conducted in the future.

Since there are a large variety of serotypes of *Salmonella*, and related reservoirs and transmission pathways could differ between serotypes (7, 41), it is of significance to continuously monitor the prevalence of *Salmonella* serotypes globally and locally. We report this distribution in Florida based on WGS data during 2017–2018. More detailed serotype sequence characterizations need further investigation. The sheer number of cases reported in Florida make it impossible to sequence all the salmonellosis cases reported to FDOH. A relevant question was therefore how representative the sequenced isolates were for all reported cases. Our results suggested that the number of laboratory-sequenced isolates was significantly associated with the reported case number but also with median income and population density per county. However, the predictive value of these two additional variables was much lower than the number of reported cases. As discussed above, enteric illnesses including salmonellosis disproportionately affect young children. When fitting the regression model, we also included the proportion of children under 5 years old in the whole population as a predictor in the initial model. This indicator did not fit significantly in the model, suggesting the distribution of sequenced isolates was not biased by the influence of age group. We therefore conclude that the lab-sequenced isolates are a relatively unbiased sample of all reported cases.

## Data Availability Statement

The datasets presented in this study can be found in online repositories. The names of the repository/repositories and accession number(s) can be found below: https://www.ncbi.nlm.nih.gov/, PRJNA230403. Additional data have been deposited in Harvard Dataverse: doi: 10.7910/DVN/KBZLMV.

## Ethics Statement

The studies involving human participants were reviewed and approved by University of Florida Institutional Review Board. Written informed consent from the participants' legal guardian/next of kin was not required to participate in this study in accordance with the national legislation and the institutional requirements.

## Author Contributions

XL, NS, and AH conceived this manuscript. JB led the laboratory sequencing work. JD led the surveillance work. XL and NS analyzed the data. XL, NS, EB, and AH wrote the manuscript. All authors contributed to the article and approved the submitted version.

## Conflict of Interest

The authors declare that the research was conducted in the absence of any commercial or financial relationships that could be construed as a potential conflict of interest.
