# Supplementary material for: Spatial Epidemiology of Salmonellosis in Florida, 2009–2018
Source: Front Public Health. 2021 Jan 11;8:603005. doi: 10.3389/fpubh.2020.603005 (PMC7931371; doi:10.3389/fpubh.2020.603005)
Supplement: Supplementary file 1 [file Data_Sheet_1.PDF]

# Supplementary Materials

## FDOH WGS Data Generation

BPHL in Jacksonville, FL started generating WGS data in 2016 for surveillance purposes, following a standardized sequencing protocol as follows: isolates were grown on a trypticase soy agar plates with 5% sheep blood (Remel, Lenexa, KS, USA). DNA extraction was conducted using the Qiagen DNeasy Blood & Tissue Kit (Qiagen, Hilden, Germany). DNA libraries were constructed using Nextera XT DNA Library Prep Kit (Illumina, Inc., San Diego, CA, USA). The genomic libraries were sequenced on the Illumina MiSeq system using a combination of v2 (500 cycle) and v3 (600 cycle) chemistries. All sequenced data are shared with CDC for surveillance purposes. These data are submitted to the NCBI BioSample database (https://www.ncbi.nlm.nih.gov/biosample/), under Bioproject number PRJNA230403 with limited metadata information.

Table S1. Over-dispersion checking of fitted negative binomial model

| Person's $\chi^2$ | Dispersion ratio |
|---|---|
| 162.85 | 1.27 |

Table S2. Multicollinearity identification of explanatory variables

| | Confirmed case | Population density | Median income | % population children under 5 | % population adults 65+ |
|---|---|---|---|---|---|
| VIF | 1.83 | 1.77 | 1.12 | 3.66 | 3.57 |

Table S3. Parameter estimation of fitted negative binomial model

| | Estimate | Std. Error | Z value | Pr (> \|z\|) |
|---|---|---|---|---|
| Intercept | -3.00 | 4.78 | -6.28 | < 0.0001 |
| Confirmed case | 0.005 | 0.0006 | 8.55 | < 0.0001 |
| Population density | 0.0006 | 0.0002 | 4.02 | < 0.0001 |
| Log(Median income) | 2.56 | 0.424 | 6.02 | < 0.0001 |
| % population children < 5 years | 0.46 | 0.15 | 3.101 | 0.0026 |
| % population adults 65+ years | 0.06 | 0.02 | 3.49 | 0.0006 |
| Log-likelihood | - 400.92 | | | |
| AIC | 815.84 | | | |
| BIC | 836.12 | | | |
| RMSE[¥](LOO[§] cross-validation average) | 20.03 | | | |

[¥]Root Mean Squared Error

[§]Leave one out

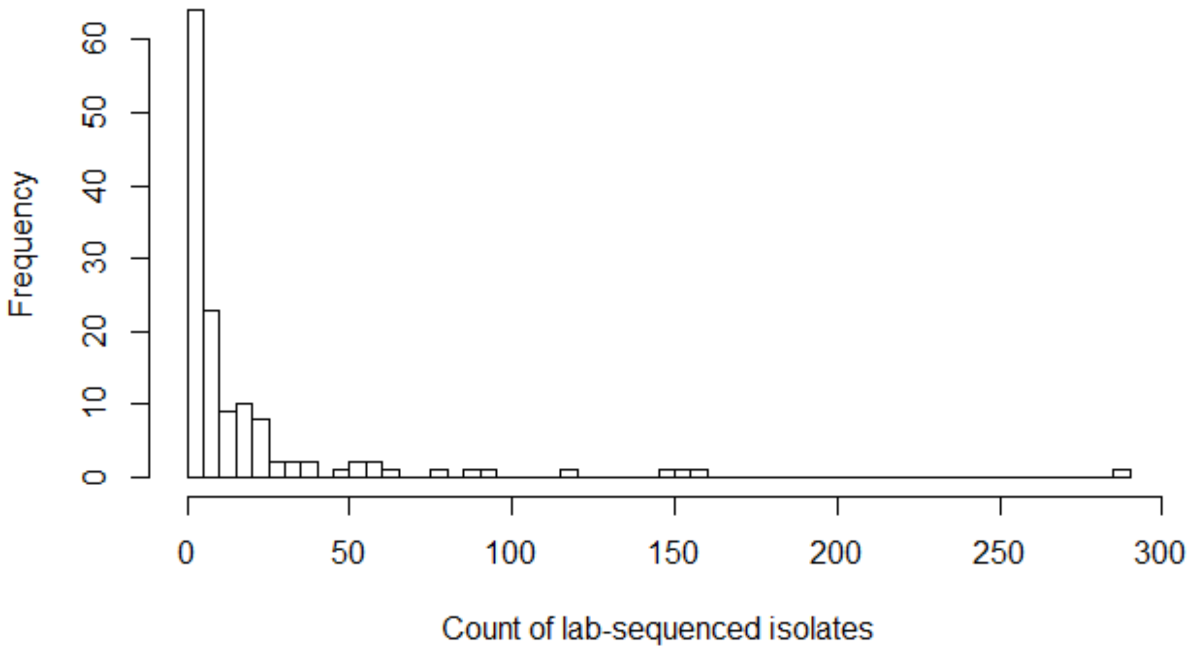


Figure S1. Frequency distribution of number of lab-sequenced salmonellosis isolates in Florida, 2017 – 2018

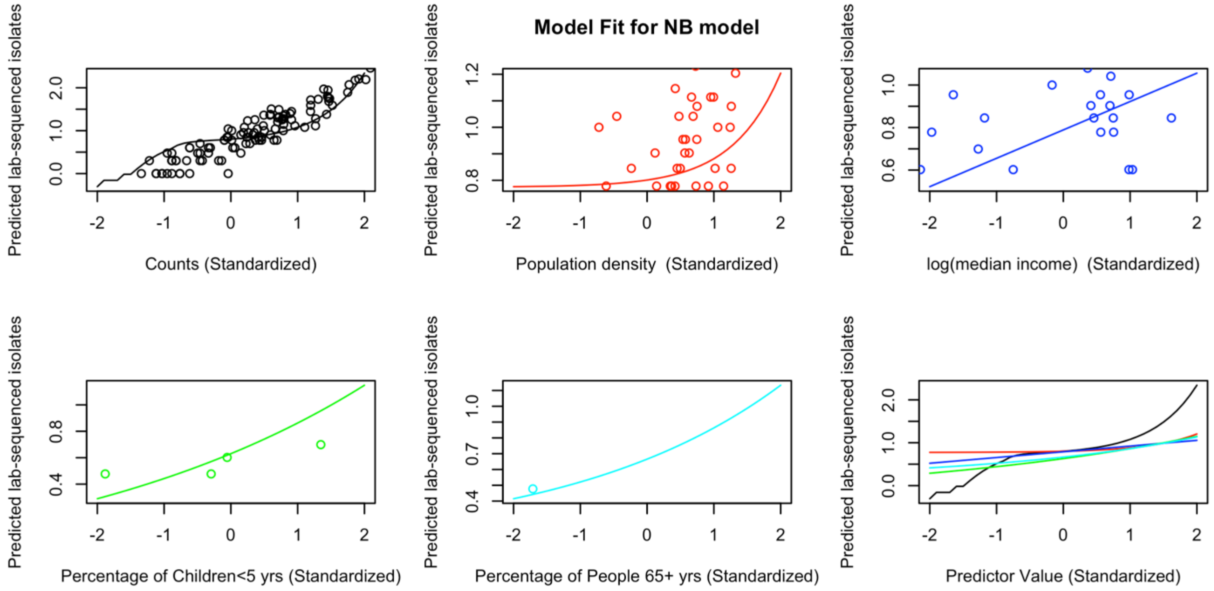


Figure S2. Negative Binomial model fit with standardized Predictor data plotted at the scale of -2 to 2, *First five plots indicate the individual independent variable predictions and last plot shows the all predictors in one plot. Lines shows the model predicted values while dots shows the observed data. (standardized to scale with same color coding for lines and dots; Black: Counts, Red: Population Density, Blue: Log(median income), Green: Percentage of children population under 5 years of age, Cyan: Percentage of population older than 65years of age)*

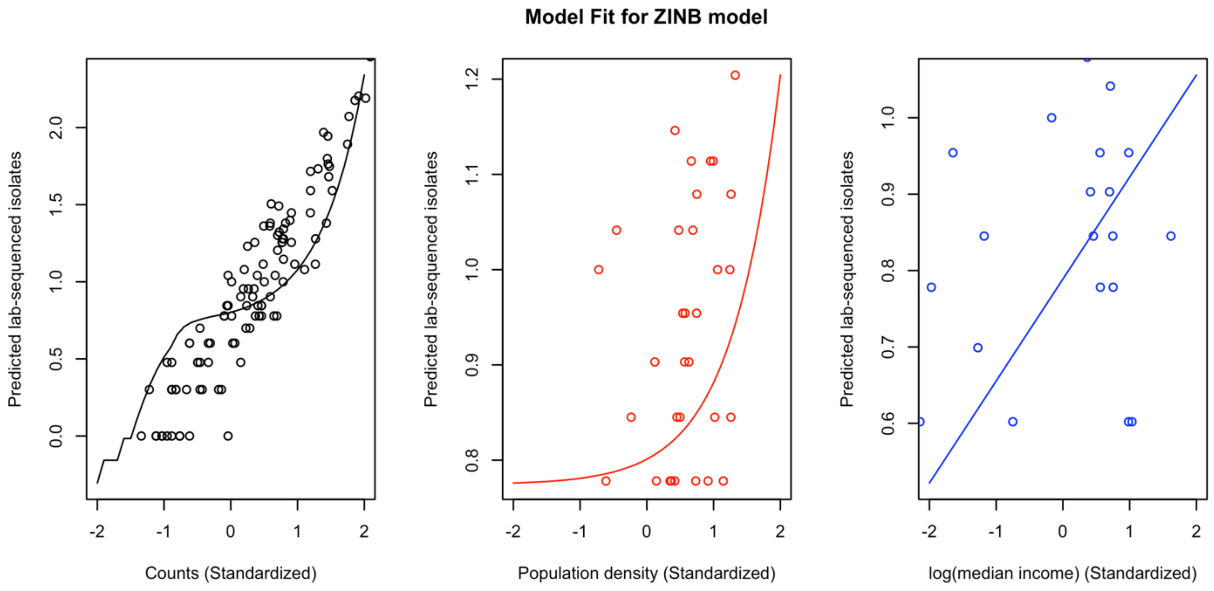


Figure S3. Zero Inflated Negative Binomial model fit with standardized Predictor data plotted at the scale of -2 to 2, *Lines shows the model predicted values while dots shows the observed data. (standardized to scale with same color coding for lines and dots; Black: Counts, Red: Population Density, Blue: Log(median income))*
